# Development, Physicochemical Characterization and In Vitro Anti-Inflammatory Activity of Solid Dispersions of α,β Amyrin Isolated from *Protium* Oilresin

**DOI:** 10.3390/molecules22091512

**Published:** 2017-09-09

**Authors:** Walter Ferreira da Silva Júnior, Jonas Gabriel de Oliveira Pinheiro, Danielle Lima Bezerra de Menezes, Natan Emanuell de Sobral e Silva, Patrícia Danielle Oliveira de Almeida, Emerson Silva Lima, Valdir Florêncio da Veiga Júnior, Eduardo Pereira de Azevedo, Ádley Antonini Neves de Lima

**Affiliations:** 1 Pharmacy Department, Health Sciences Center, Universidade Federal do Rio Grande do Norte (UFRN), Natal 59012-570, Rio Grande do Norte (RN), Brazil; walterjuniornt@hotmail.com (W.F.d.S.J.); jgopinheiro@gmail.com (J.G.d.O.P.); daniellelbmenezes@gmail.com (D.L.B.d.M.); natan.farmacia@gmail.com (N.E.d.S.e.S.); 2 Department of Pharmacy, Laboratory of Biological Activity, Federal University of Amazonas, Manaus 69077-000, AM, Brazil; patt_danielle@hotmail.com (P.D.O.d.A.); eslima@ufam.edu.br (E.S.L.); 3 Department of Chemistry, Institute of Exact Sciences, Federal University of Amazonas, Manaus 69077-000, AM, Brazil; valdir.veiga@gmail.com; 4 Military Institute of Engineering, Rio de Janeiro 22290-270, RJ, Brazil; 5 Graduate Program in Biotechnology, Laureate International Universities—UnP, Natal 59056-000, RN, Brazil; azevedoep@hotmail.com

**Keywords:** α,β amyrin, pentacyclic triterpenes, solid dispersions, hydrophilic polymers, anti-inflammatory activity

## Abstract

α,β Amyrin (ABAM) is a natural mixture of pentacyclic triterpenes that has shown a variety of pharmacological properties, including anti-inflammatory effect. ABAM is isolated from Burseraceae oilresins, especially from the *Protium* species, which is commonly found in the Brazilian Amazon. This work aimed to develop solid dispersions (SD) of ABAM with the following hydrophilic polymers: polyvinylpyrrolidone (PVP-K30), polyethylene glycol (PEG-6000) and hydroxypropylmethylcellulose (HPMC). The SDs were prepared by physical mixture (PM), kneading (KND) and rotary evaporation (RE) methods. In order to verify any interaction between ABAM and the hydrophilic polymers, physicochemical characterization was performed by Fourier transform infrared (FTIR), scanning electron microscopy (SEM), powder X-ray diffraction (XRD), thermogravimetry (TG) and differential scanning calorimetry (DSC) analysis. Furthermore, an in vitro anti-inflammatory assay was performed with ABAM alone and as SDs with the hydrophilic polymers. The results from the characterization analysis show that the SDs were able to induce changes in the physicochemical properties of ABAM, which suggests interaction with the polymer matrix. In vitro anti-inflammatory assay showed that the SDs improved the anti-inflammatory activity of ABAM and showed no cytotoxicity. In conclusion, this study showed the potential use of SDs as an efficient tool for improving the stability and anti-inflammatory activity of ABAM without cytotoxicity.

## 1. Introduction

The Burseraceae family has 18 genres and around 700 species that are distributed throughout three different tribes: Canarieae, Protieae and Bursereae. This family is located at places with low altitude and without intense cold, including tropical and drought forests as well as deserts [1]. *Protium heptaphyllum*, a common tree found in Brazilian Amazon, is one of the most widely studied species. It is popularly known as *almecegueira*, *breu-branco verdadeiro*, *almecegueira-cheirosa*, *almecegueira-de-cheiro* and *almecegueiro-bravo* [2].

An oilresin is either produced when the trunk of *Protium heptaphyllum* is injured or naturally exuded. It is generally used in popular medicine for its anti-inflammatory, analgesic, expectorant and wound healing properties [3]. Several studies have shown the presence of secondary metabolites in *Protium heptaphyllum*, especially triterpenes such as α,β amyrin (ABAM) [4].

Terpenes are biologically versatile molecules consisting of subunits of isoprene, which can be classified as monoterpenes (C10), sesquiterpenes (C15), diterpenes (C20), triterpenes (C30) and tetraterpenes (C40), commonly observed as complex mixtures. α and β amyrin are two pentacyclic triterpenes that belong to the ursano (α amyrin) and oleano (β amyrin) series [5,6]. Several pharmacological studies have shown the biological activities of ABAM, such as anti-inflammatory [7], antinoceptive, hepatoprotective [8], antipruritic [9] and gastroprotective [10] activities, which make it a promising candidate for a new drug.

Despite the potential medical benefits of ABAM, its low aqueous solubility limits its use as a drug. Thus, strategies such as salt formation [11], particle size reduction [12,13,14], formation of solid dispersion [15] and complexation with cyclodextrins [16] have been used to enhance the solubility of ABAM and increase its dissolution rate.

The use of hydrophilic polymeric matrices for the development of solid dispersions (SD) with new drug candidates has been shown to be an effective alternative to enhance the drug’s pharmacokinetic properties. SD is a well stablished technique that has been widely used due to its simple and straightforward approach [17]. However, miscibility between drug and hydrophilic polymer is an important factor for both solubility enhancement and physical stability of the obtained SD [18].

Trial and error experiments govern the process of choosing the right polymeric excipients for pharmaceutical formulations, where no systematic method is available to select the most suitable functional polymer for a particular system. However, studies have shown the importance of evaluating the interaction of different drugs with individual polymers and the effect of drug–polymer ratio on the drug’s stability and dissolution rate [19,20,21,22].

Thus, this current study aimed to develop SDs of ABAM with hydrophilic polymers (PVP-K30, PEG-6000 and HPMC) with the purpose of improving the solubility and stability of ABAM, as well as to enhance its anti-inflammatory activity. In order to investigate interactions between each hydrophilic polymer and ABAM within the solid dispersions, physicochemical characterization was performed by FTIR, SEM, XRD, TGA and DSC. In addition, an in vitro study was performed with ABAM alone and as SDs using an inflammation model based on the inhibition of LPS-stimulated J774 macrophages. Finally, a cytotoxicity assay was undertaken to determine the viability of cell growth.

## 2. Results and Discussion

### 2.1. Physicochemical Characterization

#### 2.1.1. Fourier Transform Infrared—FTIR

**Figure 1:**
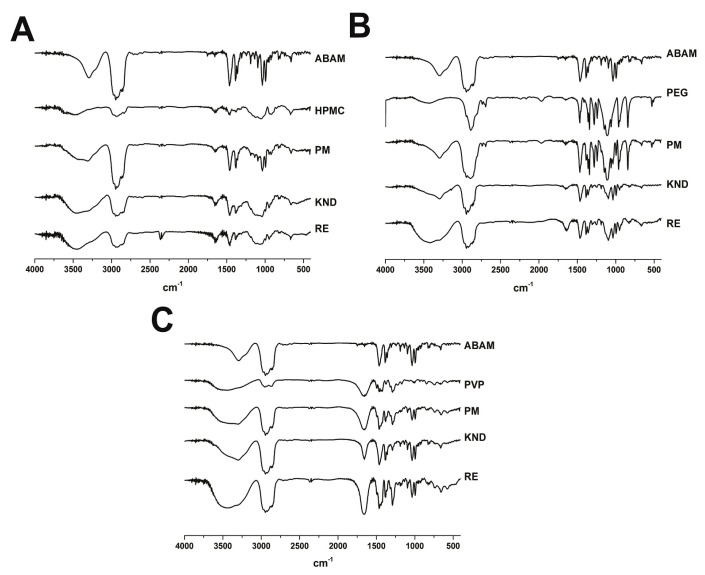
FTIR spectra of ABAM, HPMC and solid dispersions of ABAM with HPMC (**A**) FTIR spectra of ABAM, PEG-6000 and solid dispersions of ABAM with HPMC (**B**) and FTIR spectra of ABAM, PVP K-30 and solid dispersions of ABAM with PVP-K30 (**C**).

According to the FTIR spectrum for ABAM (Figure 1A), the high intensity band found at approximately 2800 cm^−1^ is associated with axial deformation of the C-H bonds in aliphatic chains. In fact, most of the ABAM structure consists of aliphatic cycloalkane chains. In addition, the band at the 3250–3400 cm^−1^ region is attributed to a hydroxyl group (O-H) linked to an aliphatic carbon chain, and the one at the 1031–1100 cm^−1^ region is due to the vibration of the C-O bond. Finally, the band at the 1400–1350 cm^−1^ region is characteristic of angular deformation of doublet germinal dimethyl and methyl groups [23,24,25].

The FTIR spectrum for HPMC (Figure 1B) shows a high intensity band in the 3443 cm^−1^ region related to the stretching of the O-H bond, as well as at 2935 cm^−1^ corresponding to the stretching of the C-H bond and at 1651 cm^−1^ and 1069 cm^−1^, corresponding to C=O bond of glucose units and C-O-C, respectively. On the other hand, the FTIR spectrum of PEG 6000 (Figure 1C) shows bands at 2980 cm^−1^, which is due to the stretching of the C-H bond and another one at 1110 cm^−1^ related to stretching of an ester bond. The spectrum of PVP K-30 (Figure 1D) reveals strong bands around 2955 cm^−1^ corresponding to the stretching of the C-H bond, as well as at 1647 cm^−1^, which is attributed to the stretching of the C-O bond. A wide band is observed at approximately 3425 cm^−1^, which is attributed to the presence of water molecules as further confirmed by thermogravimetric analysis.

When comparing the spectrum of the physical mixture of ABAM and HPMC with those of each individual compound, a spectral overlapping is observed as a result of the sum of each isolated compound. On the other hand, significant changes can be observed in the spectra of the SDs obtained through KND and RE, especially in the fingerprint region around 1500 cm^−1^. In addition, an enlargement of the band around 3250–3400 cm^−1^ and a decrease in the intensity of the one at 2800 cm^−1^ can be observed in the SD obtained by RE.

The FTIR spectra of the PM of ABAM and PEG 6000 as well as its solid dispersions obtained by KND and RE show a decrease in the band in the 3250–3400 cm^−1^ region in comparison with that of the ABAM itself, although the decrease in this band was much more intense in the solid dispersion when compared to that of the PM. In addition, the intensity of the band around 2800 cm^−1^ (characteristic of C-H bonds in aliphatic chains of ABAM) decreased in the SD prepared by KND and RE. However, no significant changes were observed between the FTIR spectrum of ABAM with that of its PM. A significant decrease in the intensity of the bands in the 1600–750 cm^−1^ region was also observed in the SD of ABAM with PEG 6000 prepared by KND and RE, whereas an increase in these bands was observed with the PM of ABAM and PEG 6000. Therefore, the most significant changes in the intensity of the bands around the fingerprint region of ABAM occurred with the SD prepared with PEG 6000 by RE, which seems to indicate a greater interaction between ABAM and PEG 6000 in the solid dispersion prepared by this method.

The FTIR spectra of the physical mixtures of ABAM and PVP K-30 as well as their solid dispersions obtained by KND and RE were also compared with that of ABAM alone. A decrease in the intensity and an enlargement of the band in the region around 3250–3400 cm^−1^ can be observed. No significant changes were observed around 2800 cm^−1^ in the PM and SD of ABAM and PVP K-30. A new low intensity band appears at the 1600–750 cm^−1^ region in the PM and SDs, which might indicate some interaction between ABAM and PVP K-30. However, significant changes occurred in the bands at the fingerprint region of ABAM when prepared as solid dispersion with PVP K-30 by RE, which seems to indicate that this method provide greater interaction between ABAM and PVP K30.

#### 2.1.2. Scanning Electronic Microcopy

**Figure 2:**
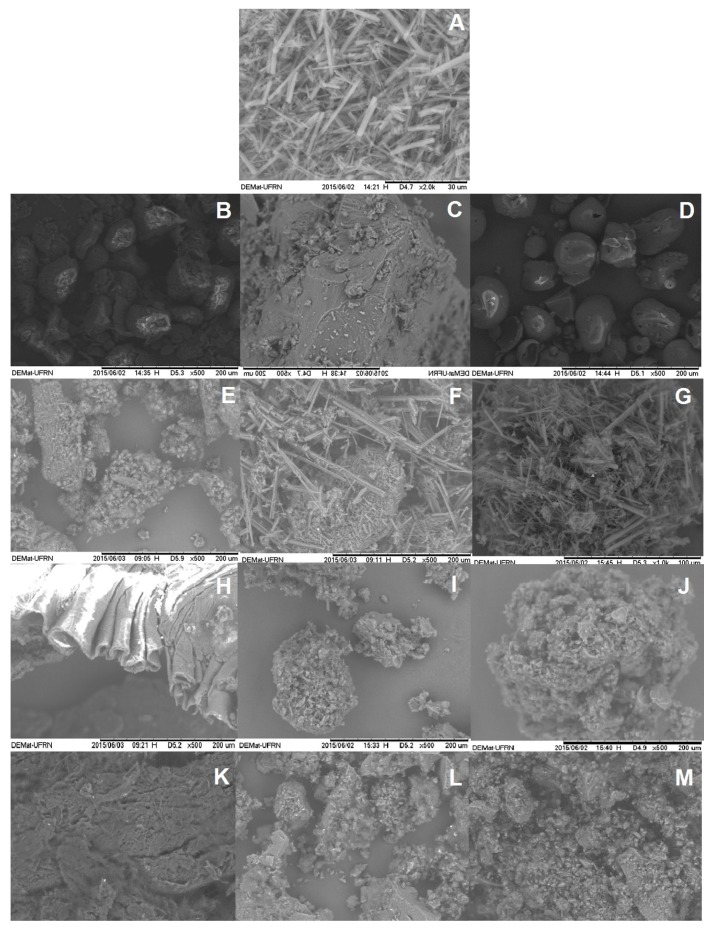
SEM micrographs of ABAM (**A**), HPMC (**B**) PEG-6000 (**C**), PVP K-30 (**D**), PMs of ABAM and HPMC (**E**), ABAM and PEG 6000 (**F**) and ABAM and PVP K-30 **(G**). SDs prepared by KND of ABAM and HPMC (**H**), ABAM and PEG 6000 (**I**) and ABAM and PVP K-30 (**J**). SDs prepared by RE of ABAM and HPMC (**K**), ABAM and PEG 6000 (**L**) and ABAM and PVP K-30 (**M**).

The SEM micrographs of the hydrophilic polymers, ABAM, PM and SDs are shown in Figure 2. The ABAM particles presented itself as geometrically irregular orthorhombic crystals. PVP K-30 (Figure 2D) showed spherical and amorphous particles, whereas PEG-6000 (Figure 2C) and HPMC (Figure 2B) particles are more porous and irregular.

The SEM pictures of the PMs of ABAM and HPMC (Figure 2E), PEG 6000 (Figure 2F), and PVP K-30 (Figure 2G) clearly show morphological heterogeneities, which seems to indicate that ABAM is simply dispersed on the polymer surface. On the other hand, the SDs prepared by KND and RE show an overall change in the morphology, evidenced by the absence of ABAM’s crystals (Figure 2H–M). The morphology of each individual component cannot be distinguished in the SDs, which might indicate that ABAM is molecularly dispersed within the polymer matrix. It is worth mentioning that a polymeric film was formed on the SDs of ABAM with HPMC prepared by KND (Figure 2H) and RE (Figure 2K), which must be due to the ability of cellulose derivatives to form films after the solvent is evaporated. This phenomenon was not observed with the other polymers.

#### 2.1.3. Powder X-ray Diffraction

**Figure 3:**
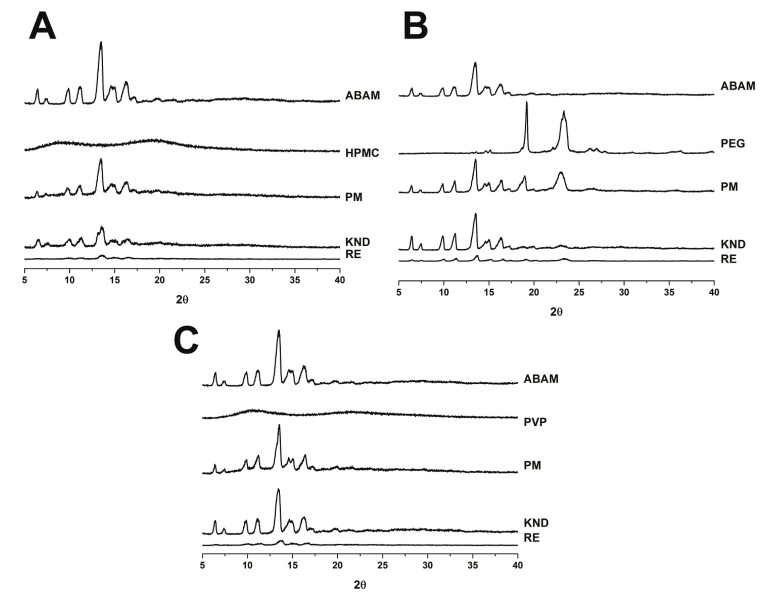
X-ray diffractograms of solid dispersions of ABAM with HPMC (**A**) PEG 6000 (**B**) and PVP K-30 (**C**).

The X-ray diffractograms of ABAM and each polymer as well as their corresponding PMs and SDs are presented in Figure 3. According to the concept of crystalline powders, these solids are characterized by three-dimensional structures that are able to diffract X-rays and exhibit a well-defined melting point [26]. The X-ray diffractograms of PVP and HPMC display a halo pattern, which is characteristic of amorphous powders [27], whereas PEG 6000 shows a diffraction pattern at 18.9° and 23°. On the other hand, intense crystalline diffraction peaks can be observed at 4° and 13° in the diffractogram of ABAM alone. In addition, other crystalline reflections of lower intensities (6°, 10°, 11°, 14° and 16°) are present in the ABAM diffractogram.

The crystallinity of ABAM particles might explain its poor aqueous solubility. Therefore, the amorphization that occurs when drugs are prepared as solid dispersions usually leads to a significant increase in the drug’s dissolution rate [28,29]. Although the crystalline reflections of ABAM were slightly reduced in the PM with HPMC, no change was observed with the PMs obtained with PVP K-30 and PEG 6000, which indicate that the PMs were not able to significantly reduce the crystallinity of ABAM. The SDs of ABAM obtained by KND with PEG 6000 (Figure 3B) and PVP K-30 (Figure 3C) slightly changed the crystallinity of ABAM, which is similar to what occurred with the PMs. The SD of ABAM with HPMC prepared by KND (Figure 3A) showed a greater reduction in ABAM’s crystalline reflections. The XRD patterns of the SDs obtained by RE show that this method is more effective in reducing the crystallinity of ABAM. On the other hand, SDs prepared with both HPMC and PVP obtained by RE significantly reduced ABAM’s crystallinity as most of its crystalline reflections almost disappeared. The SD of ABAM with PEG 6000 prepared by RE reduced the crystallinity of ABAM to a greater extent when compared to PM and KND methods; however, much of the crystalline reflection of ABAM was retained.

#### 2.1.4. Thermogravimetric Analysis

**Figure 4:**
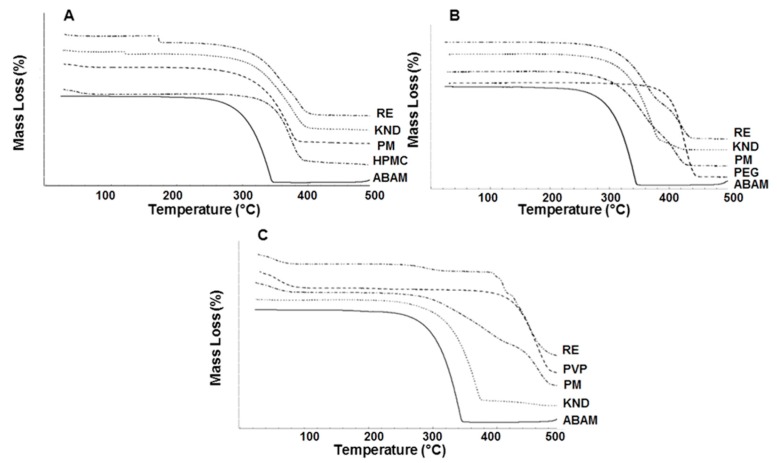
(**A**) TG curves for ABAM and HPMC alone, as well as PM and SDs prepared by KND and RE; (**B**) TG curves for ABAM and PEG 6000 alone, as well as PM and SDs prepared by KND and RE; (**C**) TG curves for ABAM and PVP K-30 alone, as well as PM and SDs prepared by KND and RE.

The thermal behavior of pharmaceuticals is crucial as it can provide important information regarding formulation stability. In addition, it may reveal incompatibilities between the drug and excipients [30]. The TG curve for ABAM shows only one well-defined stage of mass loss, which is related to its volatilization (Figure 4). The percentage of mass loss (Δm %) was around 99.5%, where the initial and final temperatures of the mass loss were 232 and 347 °C, respectively. TG results for HPMC showed high thermal stability, whose mass loss occurred in a single step, starting at 335 °C and ending at 425 °C with approximately 93% of Δm % (Figure 4A). PEG 6000 also presented a single step for mass loss (93.5%) within the temperature range of 338–448 °C (Figure 4B). TG curve for PVP K-30 shows a mass loss related to its water content (Δm = 13%), which occurred between 42 and 102 °C. Further, a main decomposition step was observed between 400 and 488 °C (68% of mass loss), followed by carbonization (Figure 4C).

In this study, all dispersed systems showed an increase in the thermal stability when compared to that of ABAM alone, suggesting that the hydrophilic polymers were able to protect this drug. For ABAM and HPMC, no difference was observed among the TG curves for PM, KND and RE. On the other hand, ABAM and PEG 6000 (Figure 4B) prepared by PM and RE methods presented higher thermal stability in comparison with that obtained by KND. Figure 4B shows that SD of ABAM and PEG 6000 showed better thermal stability than that prepared by PM. The TG curves for PM and SD prepared with PVP K-30 (Figure 4C) showed an initial mass loss due to elimination of water, which is in agreement with the TG curve for PVP K-30 alone. Moreover, samples prepared by PM and RE showed better results than that prepared by KND, where the SD prepared by RE showed the best result regarding the enhancement of the thermal stability of ABAM.

#### 2.1.5. Differential Scanning Calorimetry

**Figure 5:**
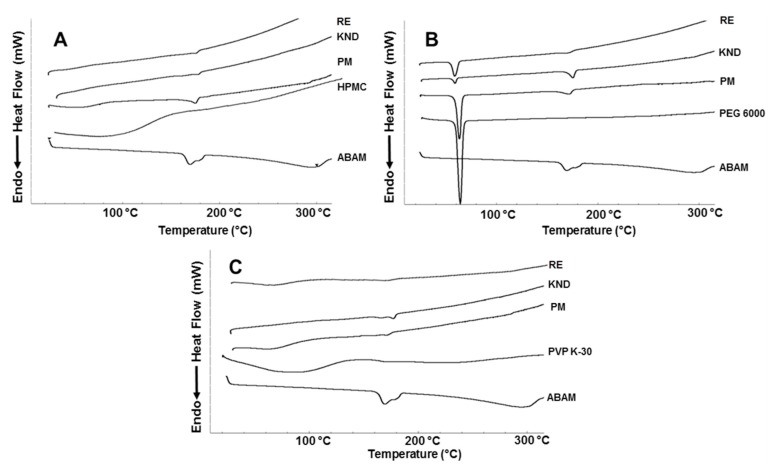
(**A**) DSC curves for ABAM and HPMC alone, as well as PM and SDs prepared by KND and RE; (**B**) DSC curves for ABAM and PEG 6000 alone, as well as PM and SDs prepared by KND and RE; (**C**) DSC curves for ABAM and PVP K-30 alone, as well as PM and SDs prepared by KND and RE.

The DSC curves for ABAM (Figure 5) show two endothermic events between 158 and 185 °C, which seem to be related to the melting of the two ABAM isomers. These two events are followed by a third endothermic event, which coincides with the weight loss due to volatilization observed in the TG analysis (Figure 4). Although PVP K-30 has several endothermic events due to the loss of water in the range of 50–120 °C (weight loss of 12.26%), no endothermic event is expected due to its amorphous characteristic. However, a melting endothermic peak around 65 °C is observed, which is probably due to loss of water. A similar profile was found with HPMC, which is another amorphous polymer, where an initial endothermic event related to water loss was also observed.

The DSC curve for PM of ABAM with HPMC shows a depression of the ABAM’s melting point, which seems be related to a partial solubilization of this drug in the polymer matrix, even though the melting event confirms the crystalline nature of ABAM in the PM. On the other hand, the SDs obtained with HPMC (KND and RE) showed an almost total suppression of the melting point of ABAM, suggesting the amorphization of the latter as well as a molecular interaction with HPMC. Regarding the systems obtained with PEG 6000, the DSC curves for the samples obtained by PM and KND show that the endothermic peaks related to ABAM’s melting still persist, confirming its crystalline character is these systems. However, the curve for the SD obtained by RE shows the complete disappearance of this endothermic event, which can be attributed to a molecular interaction between ABAM and PEG 6000, resulting in its solubilization in the PEG 6000 matrix before it reached its melting temperature. Similar finding was observed by Kumar and Mishra [31]. Samples prepared with PVP-K30 by PM and KND show DSC curves with decreased melting points for ABAM, whereas the SD prepared by RE shows no endothermic event, indicating the formation of a solid dispersion, where the drug seems to be dispersed within the polymer matrix in the amorphous state.

This phenomenon is very common when PVP is used as a polymer matrix in solid dispersions. It has been attributed to the inhibition of drug’s crystallization due to either a chemical interaction (such as hydrogen bonding between the drug molecule and the polymer) or a solvent evaporation process. In solvent evaporation methods, the slow removal of the solvent makes the system more viscous, reducing the mobility of the drug. Once the solvent is completely evaporated, the drug is frozen in the polymer matrix without forming a crystal lattice (a characteristic of the randomly ordered amorphous state) [32].

### 2.2. In Vitro Anti-Inflammatory Activity

#### 2.2.1. Quantification of Nitric Oxide in LPS-Stimulated Macrophages

Inflammation is one of the most important processes involved in the organism’s defense system, but it often progresses to chronic diseases that require pharmacological treatment. Natural products, including plant metabolites, represent great potential for the development of modern therapeutic drugs with anti-inflammatory activity [7]. Nitric oxide (NO·) is one of the major mediators of inflammation and its overproduction is associated with serious diseases such as septic shock, arthritis, stroke, chronic inflammatory conditions and autoimmune diseases. Thus, determination of the rate of NO· production in LPS-stimulated models has been widely used as an important parameter in the search for new anti-inflammatory drugs [33].

**Figure 6:**
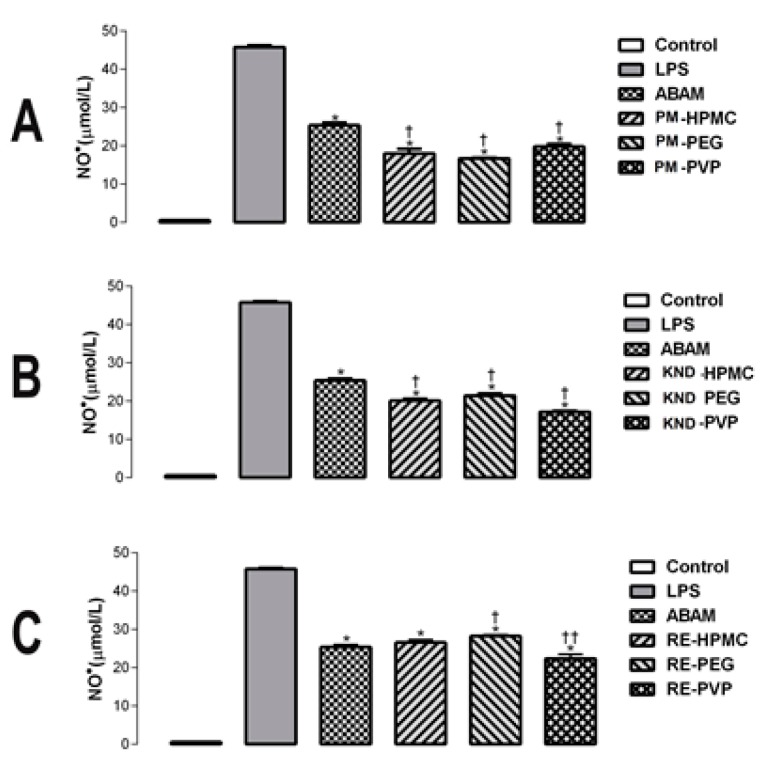
Effect of ABAM (alone and as a solid dispersion with hydrophilic polymers) on the production NO· in J774 macrophages stimulated by LPS. (**A**) NO· production in the presence of ABAM alone and as PMs compared to that of the LPS group (**B**) NO· production in the presence of ABAM alone and as a SD prepared by KND compared to that of the LPS group (**C**) NO· production in the presence of ABAM alone and as SD prepared by RE compared to that of the LPS group. Values expressed as mean ± SD (eror bar) with *n* = 5. * *p* < 0.0001, when compared to LSP group; † *p* < 0.05 or †† *p* < 0.01 when compared to ABAM and its SDs. Statistical analysis performed by one-way ANOVA followed by Newman–Keuls test.

The anti-inflammatory effects of ABAM have been largely described in different experimental models through the inhibition of release of the pro-inflammatory cytokines interleukin-1β, interleukin-6, tumor necrosis factor α, as well as the enzyme myeloperoxidase [6,9]. In this study, the effect of ABAM and its solid dispersions on the production of NO· in LPS-stimulated macrophages was investigated. The anti-inflammatory potential of ABAM and its SDs is shown in Figure 6 and in Table 1. ABAM alone and as SDs (concentration of 20 µg/mL) inhibited the production of NO· after 24 h when compared to the LPS group. The SDs decreased the production of NO· to a greater extent when compared to those of the control and ABAM alone (*p* < 0.05), indicating an improvement in the anti-inflammatory activity of ABAM when administered as SDs. It seems reasonable to assume that the solid dispersion obtained using those hydrophilic polymers is effective in enhancing the solubility of ABAM, thereby making it more bioavailable, which probably resulted in an enhanced anti-inflammatory activity.

#### 2.2.2. Cell Viability Assays

Many compounds extracted from plants can modulate cell proliferation, acting as cytotoxic agents, as is the case for antineoplastic drugs. On the other hand, they can act by stimulating the cellular growth with potential application in the wound healing [34].

**Figure 7:**
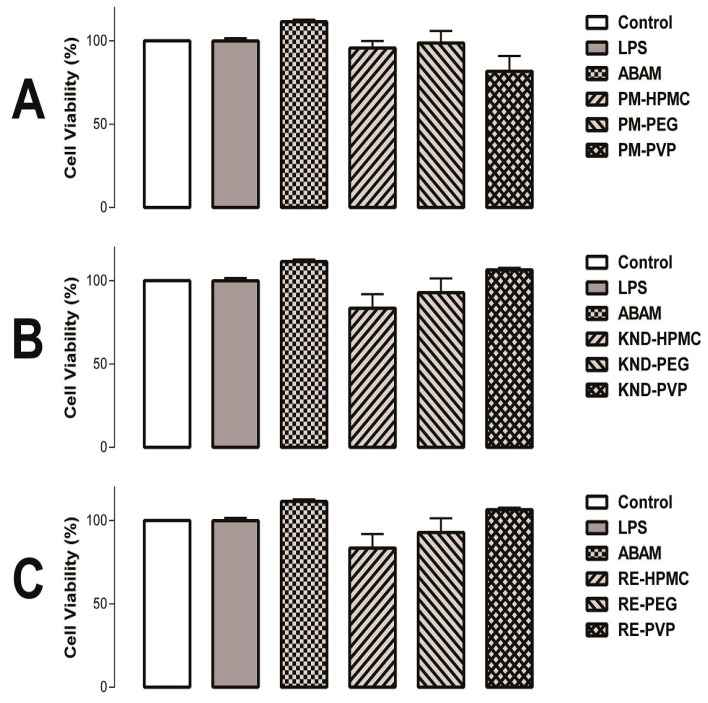
Effect of ABAM (alone and dispersed within the hydrophilic polymers) on cell viability. (**A**) Cell viability of the physical mixtures and ABAM compared to the control group; (**B**) Cell viability of ABAM and its SDs prepared by KND compared to that of the control group; (**C**) Cell viability of ABAM and its SDs prepared by RE compared to that of the control group. *p* > 0.05 when compared with the control group by one-way ANOVA followed by Newman–Keuls test.

The ability of ABAM alone as well as PMs and SDs in interfering the cell viability is shown in Figure 7. The results show that no significant difference (*p* < 0.05) was found between ABAM dispersions and that of the control group, with a cell viability higher than 80%, which classifies ABAM and its SDs as non-cytotoxic. Therefore, the results of the anti-inflammatory activity and the cell viability test indicate that a concentration of 20 µg/mL of ABAM (alone and as SDs) is effective and safe, respectively. In fact, the confirmation of low cytotoxic effect in addition to the anti-inflammatory activity is crucial during the investigation of novel compounds and new therapeutic systems as potential anti-inflammatory agents.

## 3. Materials and Methods

### 3.1. Material

ABAM was obtained from the commercial Protium oleoresins acquired in the Amazon state, AM, Brazil. PVP K30 and HPMC were purchased from Sigma Aldrich and PEG 6000 was obtained from Biotec Labmaster LTD (Pinhais, Paraná, Brazil ). The solvents used were all of analytical grade. All experiments were conducted using purified water (<1.3 μS) obtained by a reverse osmosis system, model OS50LX, Gehaka (São Paulo, SP, Brazil). All reagents were of analytical grade.

### 3.2. Preparation of SDs

Solid dispersions were prepared by physical mixture (PM), kneading (KND) and rotary evaporation (RE) methods using the weight ratio of 1:1 (ABAM:hydrophilic polymer).

#### 3.2.1. SD Obtained by PM

ABAM and the respective polymer (PVP K-30, PEG 6000 or HPMC) were precisely weighed following the 1:1 (*w*:*w*) ratio. Further, the mixtures were triturated/homogenized with mortar and pestle and stored in airtight glass desiccators under vacuum until use.

#### 3.2.2. SD Obtained by KND

ABAM and each polymer were weighted (1:1) and triturated/homogenized with mortar and pestle followed by the addition of a mixture of acetone:water (75:25, *v*:*v*). The resulting solution was dried in an oven for fifteen hours at a temperature of 60 °C and then stored in a desiccator under vacuum for future analysis.

#### 3.2.3. SD Obtained by RE

ABAM and each polymer were weighed separately (1:1, *w*:*w*) and dissolved in acetone:water (75:25, *v*:*v*) followed by evaporation under vacuum at 75 °C using a rotary evaporator Model RV 10 (GEHAKA IKA^®^, São Paulo, Brazil), operating at 150 rpm for 15 min. The resulting SD was kept in a desiccator for evaporation of the residual solvent.

### 3.3. Physicochemical Characterization

#### 3.3.1. Fourier Transform Infrared

FTIR spectroscopic analysis was performed in an IR Prestige-21 equipment (Shimadzu Corporation, Kyoto, Japan), where the samples were prepared as KBr pellets using a hydraulic press at 10 tons of pressure. The analysis was carried out in the 4000–400 cm^−1^ region with 15 scans and spectral resolution of 4 cm^−1^.

#### 3.3.2. Scanning Electronic Microscopy

The samples were mounted on aluminum stubs using double-sided adhesive tape. The morphological analysis was performed on a Hitachi TM-3000 Tabletop Microscope (Hitachi Ltd., Tokyo, Japan) at a magnification of 500×. The SEM images were obtained at an accelerating potential of 15 kV under reduced pressure.

#### 3.3.3. Powder X-ray Diffraction

Powder X-ray diffraction (PXRD) analysis were carried out using a Bruker D2 Phaser (Bruker Corporation, Billerica, MA, USA), with CuKα radiation (λ = 1.54 Å) at a voltage of 30 kV and a current of 15 mA using a Lynxeye detector. Samples were scanned at room temperature over a period of 2 h at a range of 5–40° at 0.05°/s.

#### 3.3.4. Thermogravimetry

TG thermograms were obtained using a TGA-50 Shimadzu^®^ (Tokyo, Japan) in the temperature range of 30–600 °C, using alumina crucibles with approximately 2 mg of samples under dynamic nitrogen atmosphere (50 mL·min^−1^) and heating rate of 10 °C·min^−1^. TG/DTG was calibrated using CaC_2_O_4_∙H_2_O according to ASTM standards.

#### 3.3.5. Differential Scanning Calorimetry

DSC thermal analysis was carried out in a DSC-50 cell Shimadzu^®^ (Tokyo, Japan) using approximately 2 mg of sample in aluminum crucibles under dynamic nitrogen atmosphere (50 mL·min^−1^) and heating rate of 10 °C∙min^−1^ at a temperature range of 30–500 °C. The temperature and heat flow of the DSC instrument was calibrated with indium (melting point = 157.5 °C and ∆H = 26.7 J·g^−1^).

### 3.4. In Vitro Anti-Inflammatory Study

In order to assess the production of nitric oxide (NO) by LPS-stimulated J774 macrophages, nitrite levels in culture medium was determined by Griess reaction. Cells were cultured in 24-well culture plates at a density of 1 × 10^6^ cells/mL and incubated at 37 °C in a 5% CO_2_ atmosphere for 2 h for the adhesion of macrophages. Cells were stimulated with 1 µg/mL of LPS for 60 min. After stimulation, cells were obtained with ABAM alone and as solid dispersions at a concentration of 20 µg/mL. The culture was again incubated at 37 °C at a 5% CO_2_ atmosphere for 24 h, where the supernatant was then collected for quantification of NO.

#### 3.4.1. Quantification of Nitric Oxide (NO)

Inhibition of nitric oxide production was determined by measuring nitrite levels in the supernatant of the LPS-stimulated J774 macrophage cells. NO, determined as the accumulation of nitrite (NO^2−^) in the supernatant, was measured spectrophotometrically using Griess reagent [35,36,37]. In 96 well-plates, 50 μL aliquots of cell supernatants were mixed with 100 µL of Griess reagent (1:1 mixture of 1% sulfanilamide dihydrochloride and *N*-[1-naphthyl]-etilenodiamine 0.1%), followed by incubation for 10 min at room temperature and protected from light. The absorbance at 570 nm was determined spectrophotometrically in a micro-plate reader (Beckman Coulter, Inc., Brea, CA, USA).

#### 3.4.2. Cell viability Assay

The viability of cell growth was determined using the 3-(4,5-dimethylthiazol-2-yl)-2.5-diphenyl-2*H*-tetrazolium bromide (MTT) assay. Briefly, for all experiments, 100 μL of cells was seeded in 96-well plates (3.4 × 10^3^ cells/well). After 24 h, ABAM alone and as solid dispersions (20 µg/mL) was added to each well, where the cells were incubated for 24 h. At the end of incubation time, the plates were centrifuged and the medium was replaced by fresh medium (150 μL) containing MTT (Sigma, St. Louis, MO, USA, 0.5 mg/mL). Four hours later, the formazan product was dissolved in 150 μL DMSO and the absorbance was measured at 540 nm using a multiplate reader (DTX 880 Multimode Detector, Beckman Coulter Inc., Fullerton, CA, USA). Each individual measurement was performed in triplicate.

### 3.5. Statistical Analysis

Results are presented as mean ± standard error. Two-way ANOVA with Newman-Keuls post-test was performed using GraphPad Prism version 5.00 (San Diego, CA, USA). *p*-Values less than 0.05 were considered statistically significant.

## 4. Conclusions

In this study, the physicochemical characterization indicated that ABAM was successfully prepared as amorphous solid dispersions with HPMC, PEG 6000 and PVP-K30. The decreased crystallinity of ABAM in the solid dispersions, as confirmed by XRD and DSC analysis, indicates the formation of eutectic mixtures, where the RE method seems to be the one that caused the most significant amorphization. ABAM and its SDs were shown to be safe as they did not cause a significant reduction in the viability of J774 macrophages after 24 h of treatment. Both ABAM alone and as SDs inhibited NO· production when compared to the LPS group, showing its anti-inflammatory potential. Further studies need to be carried out in order to investigate the influence of a reduction in ABAM’s crystallinity on its aqueous solubility.

## Figures and Tables

**Table 1:** Inhibition of LPS-induced NO· production from J774 macrophages by ABAM alone and as SDs.

Sample	Concentration (μg/mL)	NO^−^ Concentration	% Inhibition
LPS	1	45.75 ± 0.855	0
ABAM	20	25.37 ± 1.237	44.55
PM-HPMC	20	17.99 ± 2.037	60.68
PM-PEG	20	16.70 ± 0.200	63.50
PM-PVP	20	18.81 ± 1.114	58.88
KND-HPMC	20	20.06 ± 1.195	56.15
KND-PEG	20	21.41 ± 1.437	53.20
KND-PVP	20	17.16 ± 0.830	62.49
RE-HPMC	20	26.53 ± 1.425	42.09
RE-PEG	20	28.20 ± 0.762	38.35
RE-PVP	20	22.26 ± 2.306	51.13
